# Evaluating the reliability and quality of lupus nephritis educational content on TikTok and Bilibili: a cross-sectional content analysis

**DOI:** 10.1136/lupus-2025-001912

**Published:** 2026-09-22

**Authors:** Junjie Chen, Tianshu Wang, Ming Li, Shuangshuang Shang, Lili Cheng, Zhongfu Tang, Chuanbing Huang

**Affiliations:** 1 Anhui University of Chinese Medicine, Hefei, China; 2 The First Affiliated Hospital of Anhui University of Chinese Medicine, Hefei, China; 3 Center for Xin’an Medicine and Modernization of Traditional Chinese Medicine of IHM, The First Affiliated Hospital of Anhui University of Chinese Medicine, Hefei, China

**Keywords:** Lupus Nephritis, Autoimmune Diseases, Prevalence

## Abstract

**Objective:**

To analyse the content distribution and quality of lupus nephritis (LN)-related short videos on TikTok and Bilibili, compare differences across uploader types and examine the relationship between user engagement metrics and information reliability, thereby providing evidence to improve digital health communication strategies.

**Methods:**

This cross-sectional study screened 250 LN-related videos from TikTok (n=143) and Bilibili (n=107). Basic video characteristics and uploader types were recorded. The Global Quality Score (GQS), modified DISCERN (mDISCERN; DISCERN, a validated instrument for evaluating the reliability of health information) and a content completeness instrument were used to assess video quality. All videos were independently evaluated by three senior rheumatology clinicians. Spearman correlation analysis was applied to evaluate associations between engagement indicators and quality scores.

**Results:**

User engagement indicators (likes, comments, shares and favourites) and the proportion of professional uploaders were significantly higher on TikTok than on Bilibili (p<0.05). Although Bilibili videos were significantly longer, TikTok videos provided more comprehensive coverage of clinical manifestations and treatment (p<0.05). No significant difference in overall GQS was found between platforms (p>0.05), whereas TikTok achieved a slightly higher mDISCERN score (p<0.05). Professional institutions significantly outperformed non-professional uploaders across all quality indicators (p<0.05). Despite strong positive correlations among engagement indicators, major popularity metrics showed limited alignment with scientific quality scores.

**Conclusion:**

The quality of LN-related information on short-video platforms varies widely, with popularity metrics poorly reflecting scientifically assessed content quality. While TikTok shows a slight advantage in information reliability, both platforms require improvement in evidentiary support and content completeness. Professional institutions consistently produced higher-quality content, underscoring the need for broader participation by authoritative medical sources. A multistakeholder governance framework emphasising professional input, content quality standards and algorithm optimisation is essential to foster a trustworthy digital health ecosystem.

**WHAT IS ALREADY KNOWN ON THIS TOPIC**

- Lupus nephritis (LN) is a severe complication of SLE requiring complex, long-term management, which drives an urgent patient need for accessible information. While social media, particularly short-video platforms like TikTok and Bilibili, has become a dominant channel for health communication, existing literature across various specialties consistently highlights that online health content is often of variable quality and uneven in evidentiary support. Although the risks of low-quality information are well-documented, the specific landscape and reliability of LN-related content on major Chinese video platforms remain underexplored.

**WHAT THIS STUDY ADDS**

- This study presents the first systematic comparison of LN educational content on China’s dominant short-video platforms, TikTok and Bilibili, revealing distinct ecosystem differences where TikTok demonstrates slightly higher information reliability and user engagement. Crucially, the findings identify limited alignment between popularity metrics (likes and shares) and scientifically assessed content quality, suggesting that platform engagement may not closely reflect informational reliability. Furthermore, the analysis quantitatively confirms that content from professional institutions significantly outperforms that of other uploaders, underscoring the importance of broader participation by authoritative medical sources on these platforms and the potential value of layered educational models that link short-form content to validated external resources.

**HOW THIS STUDY MIGHT AFFECT RESEARCH, PRACTICE OR POLICY**

- This study informs research, clinical practice and policy by establishing a replicable framework for cross-platform digital health evaluation and highlighting the need to better understand the relationship between content popularity and medical quality. Clinically, findings support the development of layered educational approaches in which short-form videos serve as accessible entry points while directing patients towards validated resources such as professional society guidance and hospital-based educational materials, rather than relying solely on algorithmic recommendations. The study also recognises the complementary value of personal narrative content in providing psychosocial support for patients with chronic disease. From a policy perspective, the results advocate for a multistakeholder governance model encouraging platforms to consider content quality standards and for public health authorities to invest in high-quality content creation to strengthen the visibility of evidence-based materials in the digital ecosystem.

## Introduction

 Lupus nephritis (LN) affects 50–60% of SLE patients, with 10–20% progressing to end-stage renal disease within a decade despite therapeutic advances.^1–3^ This progression substantially increases mortality risk and imposes severe socioeconomic burden due to protracted treatment, frequent hospitalisations and potential renal replacement therapy.^4 5^ Effective patient education has been demonstrated to enhance disease understanding, treatment adherence and clinical outcomes, making improved disease awareness crucial for early identification and standardised management.^6^

The contemporary health information landscape has been fundamentally transformed by short-video platforms such as TikTok and Bilibili. Leveraging massive user bases and sophisticated algorithmic amplification, these platforms have democratised access to medical knowledge at an unprecedented scale.^7^ However, this democratisation introduces significant challenges regarding content quality and reliability.

Cross-sectional analyses across multiple specialties including rheumatology (SLE), oncology (prostate cancer), gastroenterology and endocrinology reveal a concerning dichotomy: health-related videos frequently achieve high engagement metrics yet consistently score poorly on validated quality assessments.^8–10^ This proliferation of suboptimal content creates a digital ecosystem characterised by misinformation, anecdotal evidence and unsubstantiated recommendations, potentially undermining evidence-based self-management and precipitating adverse health outcomes. Critically, while these risks are increasingly recognised in other domains, the digital information environment surrounding LN—a condition with particularly vulnerable patient populations facing severe and irreversible outcomes, remains systematically unexplored, leaving high-risk patients exposed to unvetted online information.

This study systematically evaluates LN-related content quality and reliability on TikTok and Bilibili using validated instruments (Global Quality Score (GQS), modified DISCERN (mDISCERN)), providing empirical evidence to inform collaborative strategies among medical professionals, patient organisations and platform administrators for enhancing online health information integrity and optimising patient disease management.

## Methods

### Research design

This cross-sectional study systematically evaluated LN-related video content on China’s dominant platforms: Bilibili and TikTok, using the keyword “Lupus Nephritis”. We extracted baseline characteristics and interaction metrics (likes, comments, shares, collections) to establish an empirical foundation for subsequent quality assessment and mechanistic analysis.

### Data sources and retrieval strategy

This cross-sectional study systematically evaluated LN-related videos on China’s dominant platforms: Bilibili (https://www.bilibili.com) and TikTok (Douyin, https://www.douyin.com). Using the keyword “Lupus Nephritis”, we retrieved videos via newly registered accounts without browsing history, eliminating personalised recommendation bias. Videos were ranked by platforms’ default comprehensive algorithms integrating popularity, interaction metrics and publication timing, with no temporal restrictions applied.

### Inclusion and exclusion criteria as well as screening process

**Figure 1:**
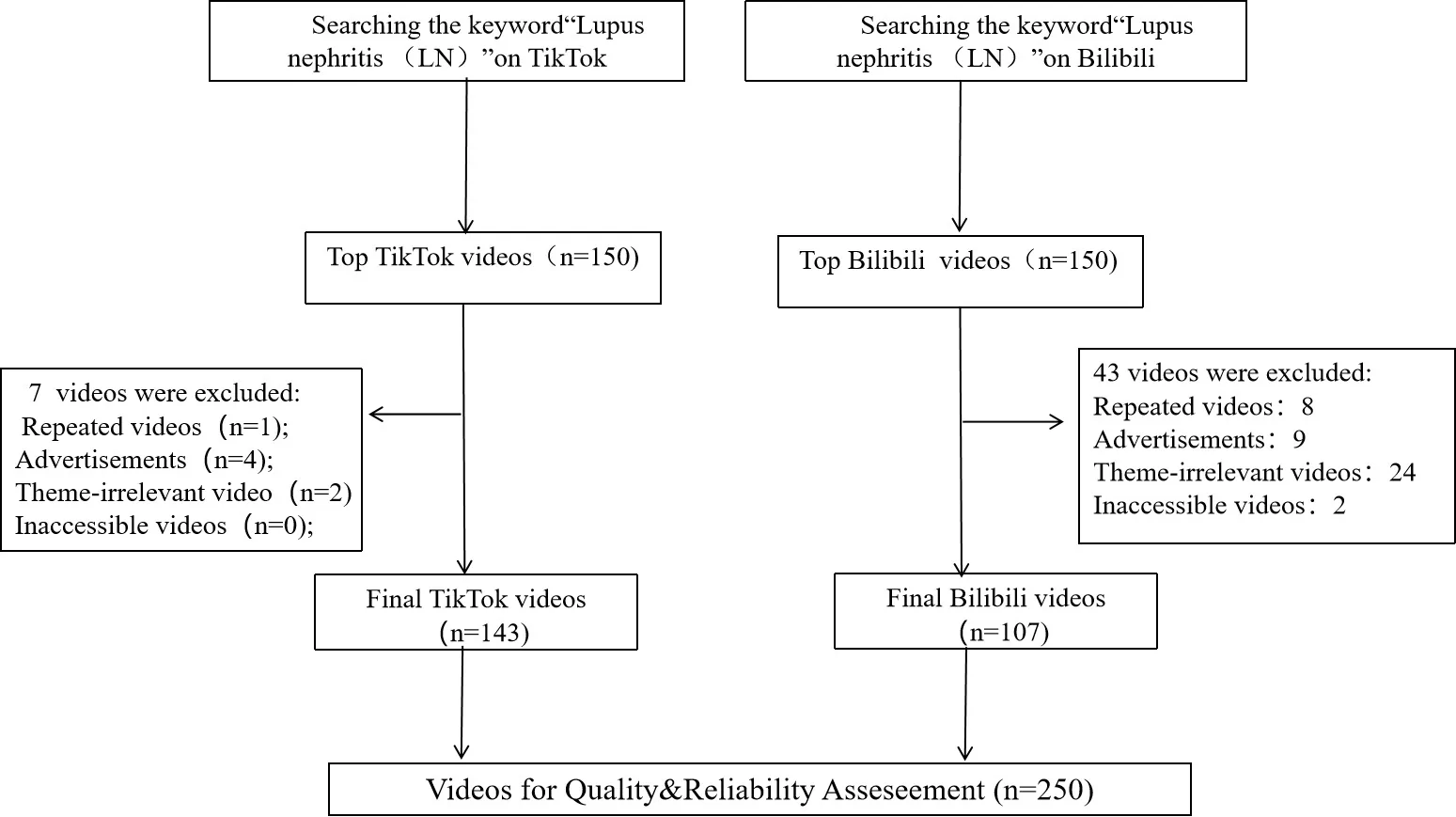
Flowchart of included videos.

The top 150 videos per platform were screened for publicly accessible, Chinese-language content representing high-exposure information sources. Exclusion criteria included: (1) duplicates; (2) commercial advertisements or product promotions; (3) videos that appeared in the search results but were not substantively related to LN; (4) videos in which LN was mentioned only briefly or superficially without meaningful educational content and (5) inaccessible content. This screening process also allowed us to assess the practical search precision of each platform under a disease-specific query. Following screening (figure 1), seven TikTok and 43 Bilibili videos were excluded, yielding a final cohort of 143 TikTok and 107 Bilibili videos.

### Data extraction

Data were automatically extracted via official platform Application Programming Interface（APIs） (cut-off: 26 October 2025) and cross-verified by two independent researchers. Variables included: (1) basic information: platform, hyperlink, title, uploader profile and duration; (2) interaction metrics: likes, comments, shares and collections. Qualitative engagement features such as comment sentiment, as well as platform metrics such as average watch duration or viewer retention, were not included because these data were beyond the scope of the present study and were not consistently accessible from the public platform interface. Videos were stratified by uploader type: professional individuals such as licensed physicians, medical researchers in rheumatology or nephrology and medical students; non-professional individuals such as patients sharing personal experiences; professional institutions such as hospitals and health organisations and non-professional institutions such as organisations or group-operated accounts without formal healthcare provider status, such as media outlets, biotechnology companies and social groups. Professional individual status was adjudicated based on publicly available account information, including platform verification labels, self-reported professional identity in the account profile, institutional affiliation when provided and the professional context presented on the account homepage. Formal offline verification of medical licences or credentials was not feasible in this study. Therefore, professional classifications were based on publicly displayed information rather than independent external verification.

We did not apply a formal bot-detection or automated-account screening procedure because the automation status of uploader accounts could not be reliably verified from publicly available platform information alone.

### Video quality assessment

Video quality was assessed using three validated instruments to capture complementary aspects of educational value. Briefly, mDISCERN was used to assess the reliability and transparency of the medical information presented, GQS was used to assess the overall educational quality, flow and usefulness of the video for patients, and the content completeness score was used to evaluate the extent to which major LN-related clinical domains were covered.

#### Modified mDISCERN instrument

The modified mDISCERN instrument was used to assess the reliability of the medical information presented in each video.^11 12^ Specifically, it evaluates whether the video is clear and understandable, cites valid sources, presents balanced and unbiased information, provides additional sources for patient reference and acknowledges areas of uncertainty. Each item was scored as yes=1 or no=0, yielding a total score ranging from 0 to 5, with higher scores indicating greater information reliability.^13 14^ See online supplemental table S1 for more details.

#### Global Quality Score

The GQS was used to assess the overall educational quality of each video, including its flow, comprehensiveness, clarity and usefulness for patients. Videos were rated on a five-point Likert scale, ranging from 1 poor quality, very limited usefulness to 5 excellent quality, very useful for patients, with higher scores indicating better overall educational value (see online supplemental table S2).

#### Content completeness assessment

Comprehensiveness was evaluated by assessing seven critical clinical domains: epidemiology, aetiology, clinical manifestations, diagnosis, treatment, prevention and healthcare using a three-level depth scale: no explanation 0 points, partial explanation 1 point and complete explanation 2 points.

### Assessment process

All included videos were independently assessed by all three senior clinicians with extensive expertise in rheumatic diseases and LN. Evaluators A (CH) and B (ML) are co-authors of this study, while Evaluator C (Xi Shen, XS) served as an independent external rater and is acknowledged in the Acknowledgements section. Evaluator A (CH) is a chief physician and professor of rheumatology at the First Affiliated Hospital of Anhui University of Chinese Medicine, with over 30 years of clinical experience in the diagnosis and management of rheumatic diseases, particularly SLE and LN. He has participated in the development of multiple national clinical practice guidelines for rheumatic diseases. Evaluator B (ML) is an associate chief physician of rheumatology at the same institution, with over 10 years of clinical experience specialising in the management of autoimmune kidney diseases including LN. Evaluator C (XS) is a chief physician of rheumatology at the same institution, with 25 years of clinical experience in autoimmune diseases including LN. Prior to formal evaluation, all three evaluators completed a standardised calibration protocol consisting of jointly reviewing a pilot subset of videos, discussing representative scoring examples for each level and aligning interpretation of the modified mDISCERN instrument, the Global Quality Score and the content completeness criteria. All videos were scored independently by the three evaluators. Inter-rater agreement was assessed on the basis of the preconsensus independent ratings. For each item, if at least two evaluators assigned the same score, that score was adopted as the final rating; in cases where all three scores differed, a consensus score was determined through discussion.

### Patient and public involvement statement

It was not appropriate or possible to involve patients or the public in the design, conduct, reporting or dissemination plans of our research. This is a retrospective analysis using publicly accessible data from social media platforms regarding LN. Due to the nature of the study using secondary, anonymised public data, no patients were directly recruited. Consequently, there was no direct patient involvement in the study design or execution.

### Statistical methods

**Figure 2:**
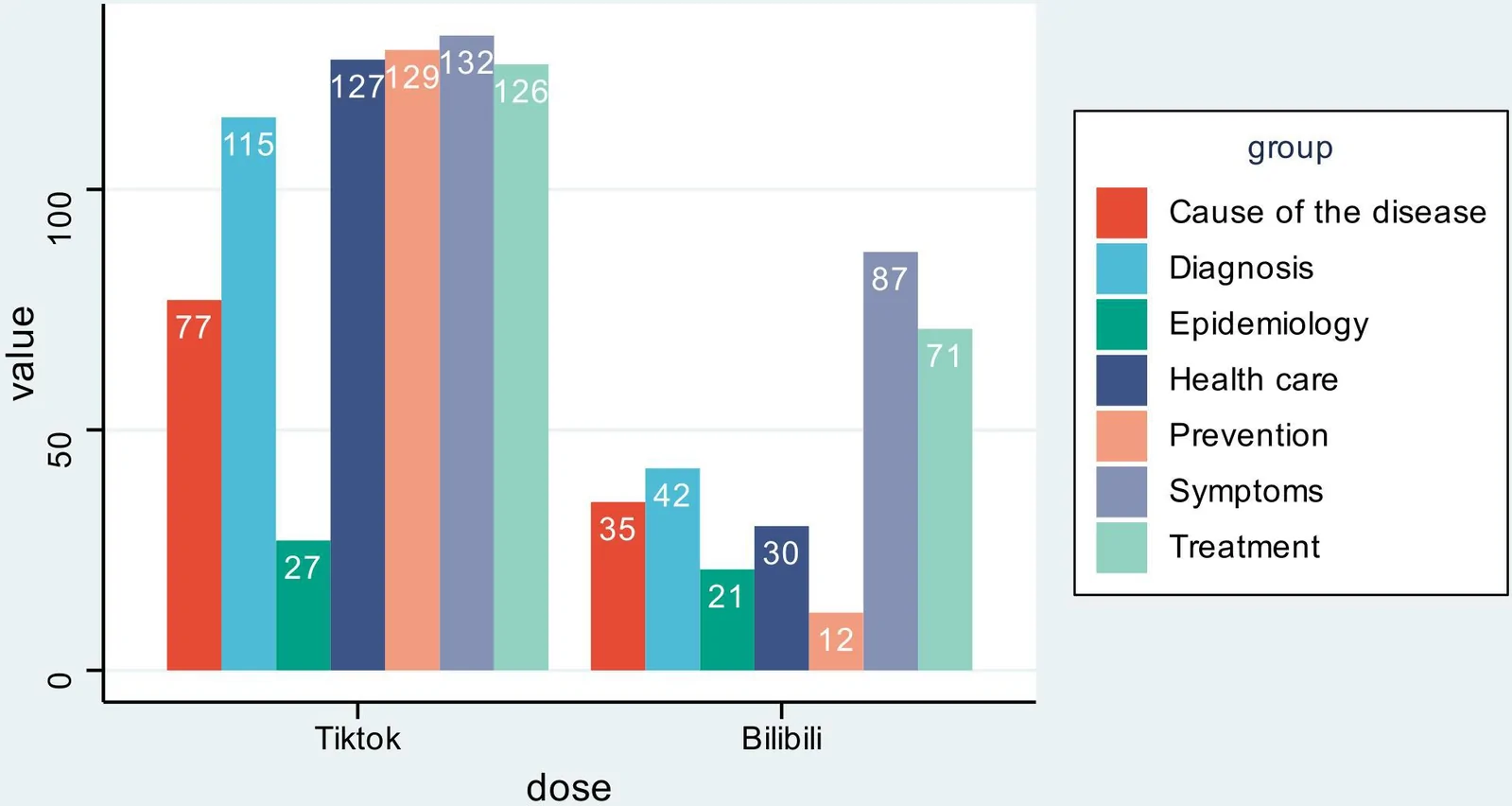
Information on the content of LN-related videos from TikTok and Bilibili. LN, lupus nephritis.

Descriptive statistics were used to summarise and analyse the dataset. All statistical computations were performed using R software (V.4.3.2). Given the non-normal distribution of the data, continuous variables are reported as medians with IQRs. Because several study outcomes such as GQS and content completeness scores were ordinal in nature, medians and IQRs were used to summarise central tendency and dispersion, and their interpretation was supported by the existing graphical displays in figure 2 and online supplemental figure S2. For comparative analyses, the Mann-Whitney U test was employed for pairwise comparisons of non-normally distributed variables between two groups.

The Kruskal-Wallis H test was used for comparisons involving three or more groups.

Spearman’s rank correlation analysis was conducted to assess the relationship between video quality scores (GQS and mDISCERN) and interaction metrics (likes, comments, shares and favourites). A two-tailed p<0.05 was considered statistically significant for all tests. Inter-rater reliability was evaluated before consensus using intraclass correlation coefficients for the total GQS score, total mDISCERN score and total content completeness score.

## Results

### Video characteristics and uploader types

During screening, excluded records mainly consisted of commercial or promotional content, videos that were retrieved by the platform search system but were not substantively focused on LN and videos in which LN was mentioned only in passing. Notably, under the same search term, Bilibili yielded a substantially larger number of excluded records than TikTok 43 versus seven, suggesting lower search precision for LN-specific educational content within the present sampling frame.

A total of 250 videos were included in the final analysis, comprising 107 (42.8%) from Bilibili and 143 (57.2%) from TikTok. Regarding interaction metrics, the TikTok group demonstrated consistently higher engagement levels compared with the Bilibili group. Specifically, the median number of likes (183 vs 10.5), favourites (42 vs 17.5), comments (15 vs one) and shares (34 vs four) were all substantially higher on TikTok. These platform-level engagement differences should also be interpreted in light of underlying differences in platform scale. Publicly available market data suggest that Douyin has a substantially larger monthly active user base than Bilibili, whereas Bilibili officially reported 376 million monthly active users and 117 million daily active users in the third quarter of 2025. Therefore, raw engagement comparisons between platforms should be interpreted with appropriate contextual caution. Conversely, regarding video duration, the Bilibili group exhibited a significantly higher median duration compared with the TikTok group (141 s vs 83 s). The detailed general characteristics of the analysed videos are presented in table 1. Because video duration was highly skewed, it was summarised using medians and IQRs rather than arithmetic means. Viewer-retention indicators such as average watch duration were not consistently accessible from the public platform interface and were therefore not included in the present analysis.

**Table 1:** General information, quality and reliability scores of lupus nephritis videos on TikTok and Bilibili

Variables	Bilibili (n=107)	TikTok (n=143)	P value
General information			
Video length(s), M (Q1, Q3)	141.00 (64.00, 634.00)	83.00 (59.00, 134.00)	<0.001
Likes, M (Q1, Q3)	10.50 (1.00, 148.25)	183.00 (93.00, 418.00)	<0.001
Collections, M (Q1, Q3)	17.50 (1.00, 110.25)	42.00 (20.50, 102.50)	<0.001
Comments, M (Q1, Q3)	1.00 (0.00, 24.00)	15.00 (5.50, 53.50)	<0.001
Shares, M (Q1, Q3)	4.00 (0.00, 30.75)	34.00 (10.50, 92.00)	<0.001
Video content			
Epidemiology, M (Q₁, Q₃)	0.00 (0.00, 0.00)	0.00 (0.00, 0.00)	0.955
Cause of the disease, M (Q₁, Q₃)	0.00 (0.00, 1.00)	1.00 (0.00, 1.00)	0.070
Symptoms, M (Q₁, Q₃)	1.00 (1.00, 1.00)	2.00 (1.00, 2.00)	<0.001
Diagnosis, M (Q₁, Q₃)	0.00 (0.00, 1.00)	1.00 (1.00, 2.00)	<0.001
Treatment, M (Q₁, Q₃)	1.00 (0.00, 1.00)	2.00 (1.00, 2.00)	<0.001
Prevention, M (Q₁, Q₃)	0.00 (0.00, 0.00)	1.00 (1.00, 1.00)	<0.001
Healthcare, M (Q₁, Q₃)	0.00 (0.00, 1.00)	1.00 (1.00, 1.00)	<0.001
Video quality			
GQS score, M (Q1, Q3)	3.00 (2.00, 3.00)	3.00 (2.00, 3.00)	0.697
mDISCERN score, M (Q1, Q3)	3.00 (2.00, 3.00)	3.00 (3.00, 4.00)	0.013

Across 250 videos, uploader distribution comprised: professional individuals (164, 65.6%), non-professional individuals (70, 28%), professional institutions (9, 3.6%) and non-professional institutions (7, 2.8%), confirming professionals’ dominant role in LN content creation. Platform-specific analysis revealed TikTok’s higher professional representation (80% professional individuals) compared with Bilibili (48% non-professional individuals, 46% professional individuals) (online supplemental figure S1).

Significant engagement variations emerged across uploader categories. Median likes differed substantially (p<0.05): non-professional institutions (1380), professional institutions (555), non-professional individuals (475) and professional individuals (193). Similar patterns appeared for favourites and shares (p<0.05), with professional institutions achieving highest favourites while non-professional creators generated higher shares. Comment volume showed no significant differences. Video duration varied significantly (p<0.05): non-professional individuals (186 s), non-professional institutions (91 s), professional individuals (83 s) and professional institutions (74 s). Detailed comparisons are presented in table 2. Table 2 should be interpreted primarily as a comparative summary of engagement heterogeneity across uploader categories rather than as a graphical display of score distributions.

**Table 2:** Characteristics of uploader types of videos on TikTok and Bilibili

Variables	Total (n=250)	Non-professional individuals (n=70)	Non-professional institutions (n=7)	Professional individuals (n=164)	Professional institutions (n=9)	Statistic	P value
Video length, M (Q₁, Q₃)	91.50 (60.00, 189.25)	186.00 (80.50, 600.25)	91.00 (62.00, 126.00)	83.00 (56.50, 136.50)	74.00 (58.00, 127.00)	χ²=22.77#	**<**0.001
Likes, M (Q₁, Q₃)	270.50 (86.75, 822.00)	475.00 (83.75, 1621)	1380.00 (971.50, 2465)	193.00 (82.75, 524.50)	555.00 (101.00, 1485)	χ²=14.50#	**0.002**
Collections, M (Q₁, Q₃)	65.50 (22.25, 232.25)	45.00 (13.25, 121.75)	59.00 (48.50, 87.50)	72.50 (27.50, 278.00)	235.00 (111.00, 538.00)	χ²=9.17#	**0.027**
Comments, M (Q₁, Q₃)	22.00 (5.00, 116.00)	19.50 (3.50, 118.25)	10.00 (4.50, 20.00)	24.00 (6.75, 111.25)	20.00 (0.00, 171.00)	χ²=2.22#	0.527
Shares, M (Q₁, Q₃)	46.00 (11.50, 177.75)	132.50 (10.75, 584.50)	672.00 (631.50, 1360.00)	33.00 (11.00, 81.25)	49.00 (19.00, 87.00)	χ²=24.48#	**<**0.001

#, χ², chi-squared test. Bold values indicate statistically significant results (P < 0.05).

M, median; mDISCERN, modified DISCERN; Q1, first quartile; Q3, third quartile.

### Video content analysis

Inter-rater agreement between all three independent evaluators was good to excellent across the three scoring systems. The intraclass correlation coefficients were 0.81 for the total GQS score, 0.83 for the total mDISCERN score and 0.88 for the total content completeness score, supporting the consistency of the evaluation process.

To evaluate content comprehensiveness, we assessed the videos across *seven* key clinical dimensions: epidemiology, aetiology, symptoms, diagnosis, treatment, prevention and healthcare. The distribution of content coverage for LN-related videos on TikTok and Bilibili is illustrated in figure 2, which provides a visual comparison of how frequently the major educational domains were addressed across platforms. In general, TikTok demonstrated broader content comprehensiveness, with a higher proportion of videos addressing these dimensions compared with Bilibili. Furthermore, the depth of explanation for each topic was systematically rated.

As presented in table 1, both platforms exhibited limited coverage of epidemiology and aetiology, with only a minority of videos briefly referencing these aspects. Because these content scores were ordinal, the reported medians and IQRs should be interpreted as summaries of relative central tendency and spread rather than equal-interval differences between score levels. Conversely, statistically significant differences were observed in the coverage of symptoms, diagnosis, treatment and healthcare management. Notably, TikTok provided significantly more comprehensive discussions on dimensions such as symptoms and treatment compared with Bilibili (p<0.05).

From the perspective of uploader categories (table 3), discussions on epidemiology were consistently limited across all groups, with no statistically significant differences observed. However, significant inter-group variations were found regarding aetiology, clinical manifestations, diagnosis, treatment and healthcare management (p<0.05). Specifically, professional individuals and institutions delivered significantly more complete and comprehensive explanations of symptoms and treatment modalities compared with non-professional counterparts. Together with figure 2, table 3 provides the detailed cross-category comparison of content completeness across uploader groups.

**Table 3:** The content characteristics of videos by different types of uploaders

Variables	Non-professional individuals (n=70)	Non-professional institutions (n=7)	Professional individuals (n=164)	Professional institutions (n=9)	Statistic	P value
Epidemiology, M (Q₁, Q₃)	0.00 (0.00, 0.00)	0.00 (0.00, 0.50)	0.00 (0.00, 1.00)	0.00 (0.00, 1.00)	χ²=1.48#	**0.689**
Cause of the disease, M (Q₁, Q₃)	0.00 (0.00, 1.00)	1.00 (0.50, 1.00)	1.00 (0.00, 1.00)	1.00 (0.00, 1.00)	χ²=9.42#	**0.024**
Symptoms, M (Q₁, Q₃)	1.00 (1.00, 2.00)	1.00 (1.00, 1.50)	2.00 (1.00, 2.00)	1.00 (1.00, 2.00)	χ²=9.70#	**0.021**
Diagnosis, M (Q₁, Q₃)	0.00 (0.00, 1.00)	1.00 (0.00, 1.50)	1.00 (0.00, 2.00)	1.00 (0.00, 2.00)	χ²=29.02#	**<**0.001
Treatment, M (Q₁, Q₃)	1.00 (0.00, 1.00)	1.00 (1.00, 2.00)	2.00 (1.00, 2.00)	2.00 (1.00, 2.00)	χ²=36.19#	**<**0.001
Prevention, M (Q₁, Q₃)	0.00 (0.00, 0.00)	1.00 (0.00, 1.00)	1.00 (0.00, 1.00)	1.00 (0.00, 1.00)	χ²=36.10#	**<**0.001
Healthcare, M (Q₁, Q₃)	0.00 (0.00, 1.00)	1.00 (0.00, 1.00)	1.00 (1.00, 1.00)	1.00 (0.00, 1.00)	χ²=34.29#	**<**0.001

#, χ², chi-squared test. Bold values indicate statistically significant results (P < 0.05).

M, median; mDISCERN, modified DISCERN; Q1, first quartile; Q3, third quartile.

### Analysis of video content quality

#### Platform comparison

Platform-level distributions of video quality scores are shown in online supplemental figure S2, which presents half-violin plots for both GQS and mDISCERN across TikTok and Bilibili. As shown in online supplemental figure S2A, the overall distribution of GQS scores was broadly similar between the two platforms, consistent with the non-significant difference in median GQS reported in table 1. As shown in online supplemental figure S2B, the distribution of mDISCERN scores was modestly shifted toward higher values on TikTok, in line with the statistically higher mDISCERN score observed for TikTok compared with Bilibili. Given the ordinal nature of these rating outcomes, the platform comparisons were interpreted in conjunction with the observed score distributions rather than on the basis of median differences alone. These visualisations complement the tabulated median and IQR results by illustrating the overall score distributions across platforms.

The assessment tools applied in this study were designed to evaluate reliability, overall educational quality and content completeness rather than to provide a separate quantitative classification of demonstrably false or dangerous misinformation.

#### Uploader-type stratification

**Figure 3:**
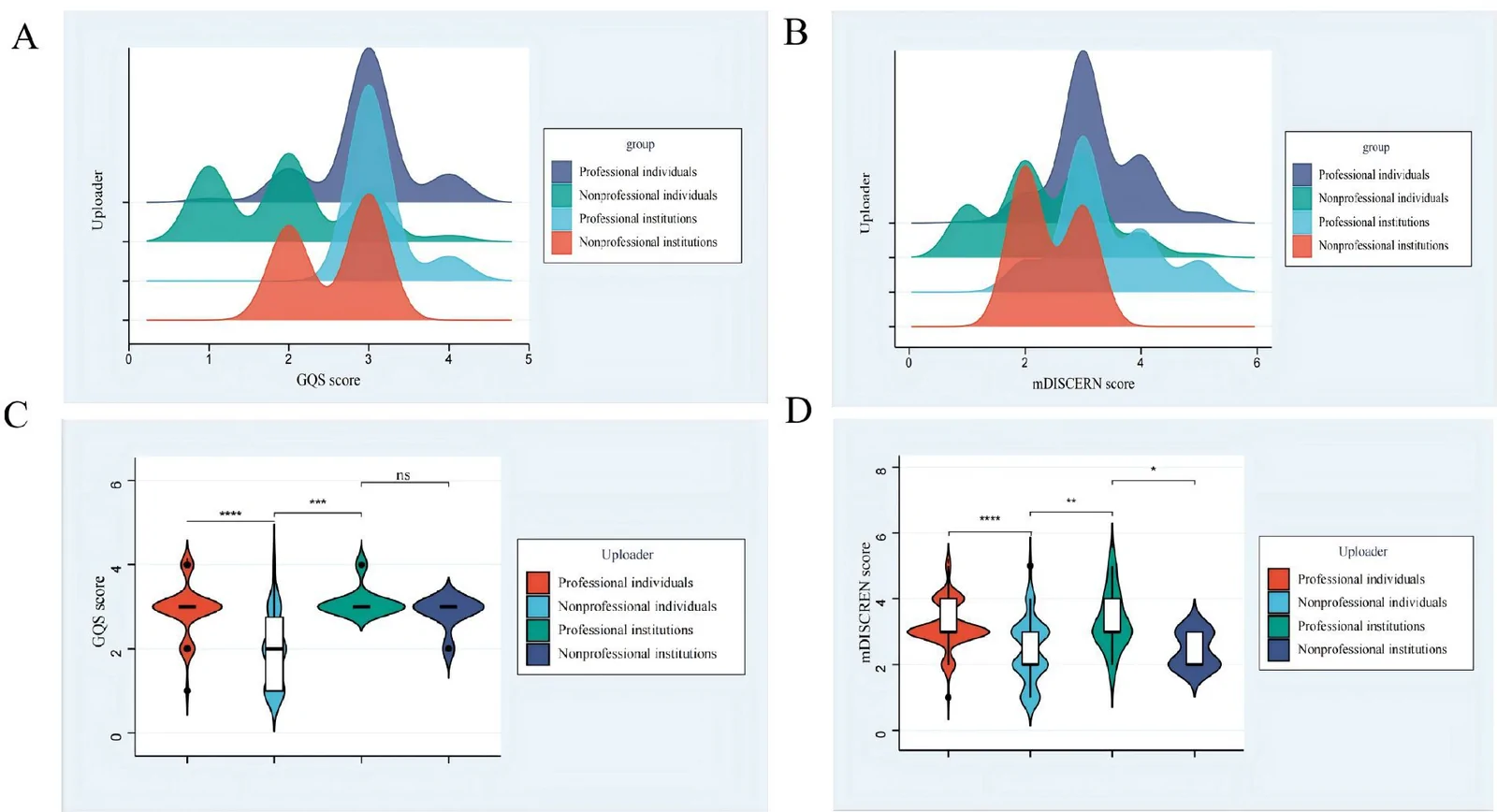
Content quality scores of videos by different types of uploaders. (A) Ridge plot of GQS scores; (B) ridge plot of mDISCERN scores; (C) violin plot of GQS scores; (D) violin plot of mDISCERN scores. GQS, Global Quality Score.

GQS density analysis revealed hierarchical performance: professional institutions (sharp, high-focused peak) > professional individuals > non-professional entities (figure 3A). For mDISCERN, professional institutions displayed a single sharp peak without tailing, indicating robust reliability. Professional individuals exhibited bimodal distribution (primary peak: 5 points; secondary: 3 points), while non-professional individuals showed bimodal trends and non-professional institutions presented broad, flat peaks at 2–3 points lacking high-score aggregation (figure 3B).

#### Violin plot analysis

Professional institutions achieved highest median GQS with narrow distributions concentrated in high-score ranges, reflecting standardised production (figure 3C). Non-professional groups exhibited wider distributions indicating heterogeneous quality (p<0.01 vs professional institutions). mDISCERN patterns paralleled GQS: professional institutions consistently scored 4–5 points; professional individuals clustered at 3–4 points; non-professional groups demonstrated significantly lower reliability with minimal high-value outliers and numerous low-value samples (<2 points), reflecting absent review mechanisms (p<0.05, figure 3D).

### Spearman correlation analysis

To investigate the relationships between various quantitative metrics of LN-related videos, specifically engagement indices (likes, shares, comments and collections), video duration, content quality (GQS) and information reliability (mDISCERN), Spearman’s rank correlation analysis was conducted.

A robust positive correlation was observed among the engagement metrics (likes, shares, comments and collections), with correlation coefficients predominantly falling within the range of 0.50 to 0.75. Conversely, video duration exhibited negligible correlation with interaction variables, demonstrating only a weak positive association with GQS and mDISCERN scores (correlation coefficients ranging from 0.12 to 0.17).

**Figure 4:**
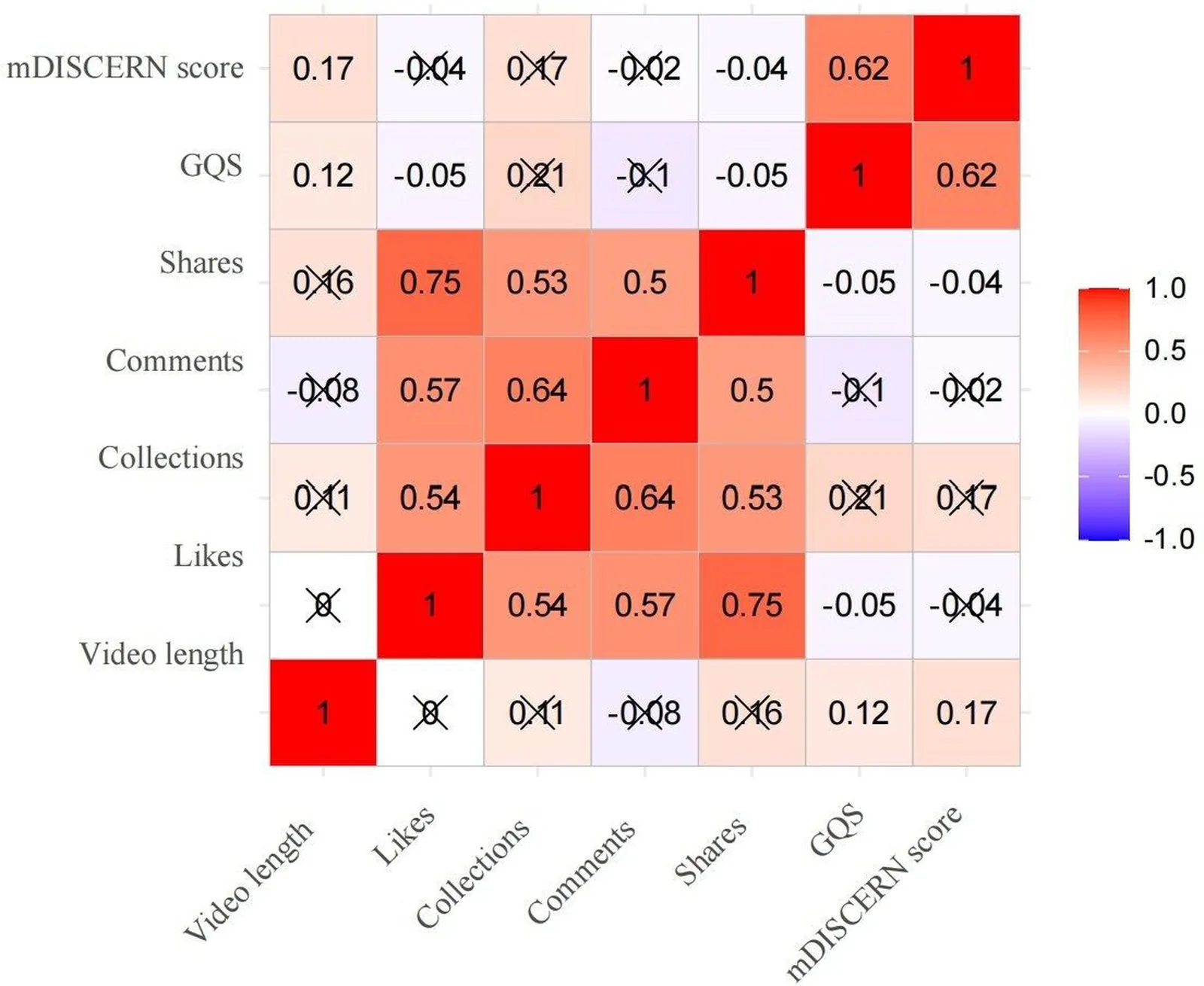
Spearman correlation analysis among different video variables, GQS and mDISCERN score concerning LN videos. GQS, Global Quality Score; LN, lupus nephritis.

Crucially, neither GQS nor mDISCERN scores showed any statistically significant correlation with audience interaction metrics (eg, likes, shares and favourites). In other words, although engagement metrics were strongly correlated with one another, their correlations with professionally assessed content quality were statistically non-significant across the main interaction indicators examined in this study. In contrast, a strong positive correlation was identified between GQS and mDISCERN scores, reflecting high internal consistency between these two distinct quality assessment tools. Detailed correlation coefficients are presented in figure 4.

This interpretation should also be considered alongside the uploader-type comparisons in table 2, which show that engagement varied across uploader categories but did not map uniformly onto the professionally assessed quality patterns observed in figure 3. Taken together, these findings suggest that audience engagement and content quality were not closely aligned in this sample.

## Discussion

In the contemporary healthcare ecosystem fully permeated by digitalisation, LN, an autoimmune disease with complex pathological mechanisms that requires long-term management,^15^ is undergoing profound structural changes in its patient education paradigms and health information access pathways.^16^ Meanwhile, social media platforms with short videos as the core carrier have increasingly evolved into the primary channel for the public, especially young people (as digital natives), to obtain health information.^17–19^

This study addresses a significant literature gap by conducting the first systematic cross-sectional evaluation of LN-related educational content on TikTok and Bilibili. Employing validated instruments (mDISCERN, GQS), we quantitatively assessed medical reliability, information quality and algorithmic dissemination patterns. Our findings reveal the current state of digital chronic disease education, providing empirical evidence to inform patient health literacy enhancement, medical provider education strategies and platform algorithm governance.

### Duality of short-video platforms in health communication for LN: digital opportunities and quality challenges

#### Correlation analysis between content quality heterogeneity and media characteristics

For chronic diseases such as SLE, particularly LN, which features complex pathological mechanisms, high heterogeneity and requires lifelong management, short-video platforms have broken down temporal and spatial barriers. They provide patients and the public with highly accessible knowledge acquisition channels, effectively alleviating the anxiety caused by information asymmetry resulting from uneven distribution of medical resources. However, the effectiveness of digital health communication depends not only on the quality of included videos, but also on how easily users can locate disease-relevant content in the first place. In the present study, the search process identified not only LN-focused educational videos, but also advertisements, promotional materials and videos that were only peripherally related to LN. Importantly, under the same keyword, Bilibili generated substantially more excluded records than TikTok, which may indicate lower search precision for LN-specific content in this setting. From a patient perspective, this means that searchability itself may shape the practical accessibility of credible health information, because users may encounter non-target or low-relevance content before reaching videos with meaningful educational value.

Despite digital media’s potential for patient education, our evaluation reveals critical quality heterogeneity even among certified medical professionals. TikTok’s GQS exhibited multimodal distribution (peaks at 2, 3 and 4 points; IQR: 2–4), reflecting ‘stratified quality solidification’ wherein traffic-oriented algorithms may simultaneously promote professionally structured medical content and more fragmented or experience-based narrative content.^20^ Conversely, Bilibili demonstrated mean reversion around 3 points, consistent with its pan-knowledge identity: extended durations enable depth but lack rigorous peer review, yielding information-rich yet qualitatively moderate content.

A pivotal and somewhat counterintuitive finding of this study is that despite the general assumption that longer formats favour medical education, TikTok achieved statistically significantly higher mDISCERN (information reliability) scores than Bilibili, although scores on both platforms remained suboptimal. Several factors may explain this disparity: first, the mDISCERN instrument prioritises clarity in treatment options, source citation and bias control. Constrained by short durations, professional creators on TikTok are compelled to condense complex LN guidelines into discrete ‘micro-learning units’. This ‘redundancy elimination’ reduces ambiguity and enforces direct, precise communication, inadvertently improving scores on specific criteria (eg, clearly stating treatment pros/cons).^21 22^ Conversely, while Bilibili’s long-form format allows for the elaboration of complex pathophysiological mechanisms, the lack of professional editing often leads to an ‘information dilution effect’. Creators may prioritise narrative case studies at the expense of systematic treatment summaries or standardised referencing, resulting in lower-than-expected structural reliability scores.

At the same time, the interpretation of quality scores should be made cautiously. The content completeness score used in this study reflects the extent to which major LN-related domains were covered, but it does not directly measure the factual accuracy of the information presented within each domain. Similarly, a GQS value clustered around the middle range should not necessarily be interpreted as indicating true equivalence in educational quality across uploader groups, because such scores may also reflect the subjective features of the instrument and the inherent constraints of short-form video in presenting complex clinical information. Future research would benefit from incorporating a clearer external benchmark, such as professional society guidelines or governmental health information sources, to improve the evaluation of accuracy and evidentiary quality.

Despite platform-specific variations, the median mDISCERN score across both platforms hovered around 3 points, suggesting that the overall reliability of platform-based LN education remains moderate rather than strong. Item-level assessment showed that source citation was present in a substantial proportion of videos. Specifically, 185 of 250 videos 74.0% cited valid sources overall, including 83 of 107 77.6% on Bilibili and 102 of 143 71.3% on TikTok (see online supplemental table S3). However, the overall mDISCERN pattern still indicated limitations in balance, completeness and the discussion of uncertainty, which may reduce the evidentiary clarity of these videos for patient education purposes. This interpretation is consistent with previous studies showing that social media health content may remain uneven in quality even when some form of source citation is present.^23 24^ For instance, studies on atopic eczema^25^ and knee osteoarthritis^2 26^ have similarly reported variable quality and limited reliability across digital platforms.

Current short-form digital content should be viewed as a complement to rather than a substitute for professional clinical education. A more practical future direction may be the development of layered educational models, in which short-form videos serve as accessible entry points while also directing users towards validated external resources, such as professional society guidance, hospital-based educational materials or other authoritative sources. In this context, standardising digital health content quality and strengthening the visibility of evidence-based materials may help improve the educational value of platform-based communication. More broadly, these findings also reflect an inherent limitation of microlearning formats: short-form videos may be effective for brief explanations, symptom awareness or introductory education, but they are less well suited to conveying the full complexity of guideline-based management for conditions such as LN.

### Information content and quality of LN-related short videos

A core finding of this study is that the predominant proportion of short videos related to LN on TikTok and Bilibili failed to meet established standards for scientific quality and reliability. This observation aligns closely with previous research investigating social media content regarding other chronic conditions. ^27–29^The short-video format inherently constrains breadth and depth: comprehensively addressing LN’s complex pathology, protean manifestations and individualised treatment within time limits proves challenging. While low GQS/mDISCERN scores do not negate clinical value, many videos provide practical guidance (managing prednisone side effects, dietary modifications). The critical concern is conflating useful lifestyle advice with unverified medical claims, creating a confusing information ecosystem undermining valid health messaging.

Despite the general assumption that long-form platforms facilitate complex information dissemination, this study found that TikTok exhibited superior structural completeness regarding clinical dimensions of LN. Statistical analysis revealed that TikTok outperformed Bilibili in covering core domains such as clinical manifestations, diagnosis, treatment and healthcare management (p<0.05). A plausible explanation is that TikTok creators often adopt a condensed ‘problem-solution’ closed-loop model, for example, ‘What is LN? → Symptoms → Treatment’, thereby ensuring broad, even if sometimes superficial, coverage. Bilibili’s long-form videos prioritise depth in specific dimensions (pathological mechanisms, personal narratives) but lack holistic coverage. Both platforms exhibit epidemiology/aetiology deficits, reflecting ‘patient-centred pragmatism’^30^: prioritising actionable symptom management over foundational science to align with traffic algorithms.^7^

Professional individuals and institutions generally provided more structured clinical discussions, which was reflected in their higher GQS and mDISCERN scores and their stronger alignment with guideline-oriented informational content. However, this should not be interpreted to mean that personal narrative videos are inherently less valuable. For patients with chronic diseases such as LN, experiential narratives may provide psychosocial support, reduce emotional isolation and help patients feel understood in ways that purely informational content may not fully achieve. In this sense, educational and personal narrative videos may serve different but complementary functions. Notably, some individual physicians scored below certain non-professional creators, likely reflecting differences in video organisation, communication style or evidentiary presentation rather than medical expertise alone. Taken together, these findings suggest the need for a more complementary communication ecosystem, in which professional sources provide structured diagnosis-related and treatment-related information, while patient communities may contribute experiential and supportive perspectives.

Addressing quality gaps requires greater participation by rheumatology, nephrology and immunology experts in the digital health communication ecosystem. For high-risk chronic conditions such as LN,^7 31^ incomplete, weakly supported or poorly contextualised information may create confusion for patients who are trying to understand symptoms, treatment options and self-management recommendations. At the same time, because the present study did not include a dedicated coding framework for demonstrably false or dangerous misinformation, these findings should not be interpreted as quantifying explicitly harmful content. Rather, they highlight the importance of stronger evidentiary support, clearer communication standards and broader participation by authoritative medical sources on short-video platforms.^32^

### Limited alignment between video quality and user engagement

This study suggests that audience engagement and professionally assessed content quality were not closely aligned in this sample. Specifically, although engagement metrics were strongly correlated with one another, their associations with GQS and mDISCERN scores were statistically non-significant. In other words, higher scientific rigour was not associated with greater popularity on the platform in our sample, although popularity itself may not fully capture a video’s clinical or behavioural influence on viewers.

Importantly, platform engagement should not be interpreted as equivalent to clinical impact. For patients with chronic and complex conditions such as LN, the practical influence of a video may depend not only on factual accuracy but also on message framing, narrative style, emotional resonance and perceived relevance. These communication features may affect whether viewers remember the message, discuss it with others, seek medical care or modify self-management behaviours. Because the present study was designed as a cross-sectional content analysis of publicly available videos, we were able to evaluate observable engagement indicators and information quality but not downstream patient-level outcomes such as comprehension, behavioural intention, treatment adherence or healthcare-seeking behaviour. Future research should therefore combine content analysis with patient surveys, experimental designs, sentiment analysis or longitudinal follow-up to better evaluate the real-world clinical impact of different messaging strategies.

This pattern is consistent with prior studies in other disease areas suggesting that audience engagement does not necessarily track professionally assessed information quality.^33 34^ This indicates that in the current algorithm-recommended ecosystem, high-quality evidence-based medical content has not been granted a level of dissemination weight that matches its value.

LN’s complex pathology may create information asymmetry in online settings, where some viewers may rely more heavily on peripheral cues^35^ such as visual appeal, narrative fluency or creator charisma than on evidentiary quality alone. In addition, treatment-related anxiety may make emotionally resonant content especially salient for some patients, including content that appears to offer simpler or more reassuring alternatives.

Furthermore, our Spearman correlation analysis shows a very strong positive correlation among likes, shares and favourites, indicating that user engagement indicators are highly interdependent on short-video platforms. A video that receives a large number of likes is more likely to be recommended to more users by the algorithm, thereby gaining more shares and favourites. This creates an amplification effect: once a video gains initial popularity, its dissemination may expand rapidly through algorithmic amplification even when viewer engagement is not aligned with professionally assessed information quality.

Addressing this requires multistakeholder action. Medical experts must adopt accessible language and visual elements (animations, charts) to enhance engagement beyond mere accuracy. Platforms should prioritise certified professionals and authoritative institutions in algorithms, proactively displaying curated ‘health topic’ collections for LN-related searches to ensure accurate knowledge dissemination.

### Robustness and innovation of the research methodology

This study employs a rigorous cross-platform design, systematically sampling TikTok and Bilibili to construct a comprehensive landscape of LN-related digital health communication while mitigating single-source selection bias. By integrating multiple validated assessment tools (mDISCERN, GQS) and analysing correlations between creator identity, algorithmic metrics and content quality, we established a multidimensional quality assessment framework to examine structural variables influencing health information dissemination effectiveness.

For LN, an autoimmune disease requiring lifelong management. Information constitutes an integral component of treatment. Short-video platforms have emerged as primary digital channels for patient self-management education.^36 37^ However, the ‘quality-traffic decoupling’ phenomenon revealed in this study demonstrates that algorithmic reality frequently diverges from clinical reality, lacking high-level evidence supporting educational effectiveness.

These findings have several practical implications for digital health communication in LN. Overall content quality remained variable across platforms and higher audience engagement did not consistently align with professionally assessed information quality. These patterns support the need for clearer quality standards, stronger evidentiary support and better linkage between short-form educational content and authoritative external resources.

In addition, the content gaps identified in areas such as daily care and aetiology suggest opportunities for more structured clinic-community-platform collaboration. Medical institutions, professional associations and patient organisations may help strengthen the digital information environment by co-producing evidence-based content addressing infection prevention, dietary management, pregnancy considerations, follow-up adherence and psychological support in forms that are accessible to patients.

In conclusion, short videos appear to play an increasingly visible role in shaping how patients encounter LN-related information online. This makes it important for clinicians, institutions and platforms to support the availability and visibility of high-quality, comprehensible and evidence-informed educational content.

### Limitations

This study has several limitations. First, the analysis was restricted to TikTok and Bilibili and relied on a single disease-specific keyword, “Lupus Nephritis”. As a result, the findings may not capture videos labelled with broader lay terms, alternative hashtags or patient-generated expressions, and the observed platform differences in search precision and content characteristics should be interpreted within the context of this query design. In addition, some videos may have represented only one segment of a multipart educational series rather than a fully self-contained unit, which may have led to underestimation of the completeness of individual videos.

Second, although modified mDISCERN, GQS^38 39^ and content completeness scoring provided a structured framework for evaluation, these tools retain subjective elements. In particular, content completeness reflects breadth of topic coverage rather than factual accuracy, and GQS ratings may also be influenced by the inherent constraints of short-form video in conveying complex clinical information.^40^ Future research could further enhance objectivity by expanding the evaluator panel and incorporating more explicit external benchmarks such as professional society guidelines or governmental health information sources.

Third, the present study did not include a dedicated coding framework to identify and quantify demonstrably false or dangerous misinformation. Therefore, we were not able to distinguish systematically between videos that were merely incomplete or weakly supported and those that contained explicitly incorrect or potentially harmful guidance.

Fourth, our analysis focused on publicly visible quantitative engagement indicators. Qualitative engagement features such as comment sentiment, as well as viewing-behaviour metrics such as average watch duration or retention, were not consistently accessible from the public platform interface. Similarly, because the study was cross-sectional and based on publicly available content, it could not evaluate patient comprehension, behavioural responses or clinical outcomes. In addition, the substantial intercorrelations among engagement metrics suggest that future research may benefit from dimension reduction techniques such as principal component analysis to generate composite engagement indices.

Future research should combine content analysis with longitudinal designs, patient-centred outcome measures, qualitative engagement indicators such as comment sentiment and viewing-behaviour metrics and computational methods to better understand the value and risks of short-video health communication in LN. Bridging the gap between professional evaluation and patient perception will be essential for future work.

## Conclusion

This study evaluated the quality and reliability of LN educational short videos on TikTok and Bilibili, revealing significant content quality variability. While professional creators (medical institutions and physicians) generally produced more reliable content, popularity metrics showed limited correlation with scientific accuracy. TikTok demonstrated marginally superior performance compared with Bilibili.

Critical information gaps were identified in diagnostic processes, treatment risks and lifestyle management, alongside uneven evidentiary support across videos. Popularity-based content ranking may complicate how patients interpret and prioritise health information online. Enhancing the visibility of high-quality, comprehensible and actionable LN educational content is essential for improving treatment adherence and health outcomes.

Addressing misinformation requires collaborative efforts among platforms, healthcare providers and users. We recommend platforms implement quality labelling systems and authoritative source prioritisation, encourage standardised professional content co-creation and deploy artificial intelligence（AI）-driven quality control mechanisms. Future research should incorporate longitudinal and cross-cultural comparative approaches to establish a more reliable digital health information ecosystem.

## Supplementary material

10.1136/lupus-2025-001912 online supplemental file 1

## Data Availability

Data are available upon reasonable request.
