# Supplementary material for: Evaluating the reliability and quality of lupus nephritis educational content on TikTok and Bilibili: a cross-sectional content analysis
Source: Lupus Sci Med. 2026 Sep 22;13(2):e001912. doi: 10.1136/lupus-2025-001912 (PMC13599989; doi:10.1136/lupus-2025-001912)
Supplement: Supplementary file 1 [file lupus-13-2-s001.docx]

# Evaluating the reliability and quality of Lupus nephritis educational content on TikTok and Bilibili: A cross-sectional content analysis

Junjie Chen ^1^, Tianshu Wang ^2^, Ming Li ^3^, Shuangshuang Shang ^3^, Lili Cheng ^2^, Zhongfu Tang ^2^, Chuanbing Huang ^3*^

**Supplementary Table S1** The Modified DISCERN (mDISCERN) quality criteria.

**Reliability Score**

1. Is the video clear, concise, and understandable?

2. Are valid sources cited?

3. Is the content presented balanced and unbiased?

4. Are additional sources of content listed for patient reference?

5. Are areas of uncertainty mentioned?

* (1 point for answer ‘yes’, 0 point for answer ‘no’)

**Supplementary Table S2** The Global Quality Score (GQS) quality criteria.

| **Item features** | **Points** |
| --- | --- |
| Poor quality; poor flow of the videos; most information missing; not at all useful for patients | 1 |
| Generally poor quality; some information listed, but many important topics missing; of very limited use to patients | 2 |
| Moderate quality; suboptimal flow; some important adequately discussed, but other information poorly discussed; somewhat useful for patients | 3 |
| Good quality and generally good flow; most of the relevant information listed, but some topics not covered; useful for patients | 4 |
| Excellent quality and flow; very useful for patients | 5 |

**Supplementary Table S3.** Proportion of videos citing valid sources according to modified mDISCERN item 2

| Platform | Videos citing valid sources, n | Total videos, n | Proportion, % |
| --- | --- | --- | --- |
| Bilibili | 83 | 107 | 77.6 |
| TikTok | 102 | 143 | 71.3 |
| Total | 185 | 250 | 74.0 |

**Figure S1** General information of LN-related videos from TikTok and Bilibili. (A) A circular pie chart showing the percentage of uploader types across all platforms; (B) a percentage stacked bar chart showing the percentage of uploader types on different platforms (TikTok and Bilibili). LN: Lupus Nephritis.

**Figure S2** Content quality scores of videos across different platforms (A: Half-violin plot of GQS scores; B: Half-violin plot of mDISCERN scores).
